# Re-Intervention Rate, Timing, and Indications Following Coronectomy of the Mandibular Third Molar: A Systematic Review of Systematic Reviews

**DOI:** 10.3390/jcm14113877

**Published:** 2025-05-30

**Authors:** Federica Di Spirito, Mario Caggiano, Alfonso Acerra, Iman Rizki, Grazia Leonetti, Gianluca Allegretti, Massimo Amato

**Affiliations:** Department of Medicine, Surgery and Dentistry, University of Salerno, Via S. Allende, 84081 Baronissi, Italy; macaggiano@unisa.it (M.C.); alfonso.acerra10@gmail.com (A.A.); i.rizki@studenti.unisa.it (I.R.); dottg.leonetti@gmail.com (G.L.); dott.allegrettigianluca@gmail.com (G.A.); mamato@unisa.it (M.A.)

**Keywords:** coronectomy, partial odontectomy, intentional root retention, root retention technique, third molar, inferior molar, wisdom tooth, wisdom teeth

## Abstract

**Background/Objectives**: Coronectomy is an alternative to complete third molar extraction to reduce the risk of inferior alveolar nerve injury. This systematic review of systematic reviews evaluates re-intervention rate, timing, and indications after mandibular third molar coronectomy. **Methods**: A systematic search following PRISMA guidelines was conducted across Scopus, MEDLINE/PubMed, BioMed Central, Web of Science, Cochrane Library and PROSPERO. Studies reporting re-intervention rates after at least six months from coronectomy were included. Data extraction focused on re-intervention timing, indications, and complications. **Results**: Six systematic reviews, including 5896 subjects and 7913 successful coronectomies (not requiring immediate tooth extractions), were analyzed. The overall re-intervention rate was 4.45%, with timing ranging from six months to ten years (mean: 10.4 months). Root exposure (16.76%) was the primary cause, followed by infection (4.55%) and pain (2.84%). Root migration (12.20%) was common, while inferior alveolar nerve injury remained rare (0.76%). **Conclusions**: Coronectomy is a viable alternative in high-risk cases, with a low re-intervention rate. Root migration and exposure require long-term follow-up. Standardized imaging protocols and refined re-intervention criteria are needed.

## 1. Introduction

The surgical extraction of third molars is one of the most commonly performed procedures in oral and maxillofacial surgery. The indications for third molar extraction vary and include impaction, pericoronitis, caries, cyst formation, and orthodontic reasons. Studies report that the prevalence of third molar extractions ranges from 35.9% to 58.7% in different populations [1,2,3].

Despite its frequency, surgical removal of mandibular third molars carries the risk of complications, including infection, alveolar osteitis, pain, swelling [4,5], as well as lingual nerve injury (LNI), and, above all, inferior alveolar nerve injury (IANI) [6]. In detail, the incidence of IANI varies from 0.4% to 8.4%, with permanent nerve damage occurring in up to 3.6% of cases [7,8,9].

To mitigate the risk of IANI, coronectomy was proposed in the 1980s [10,11] as an alternative to complete third molar removal. First described by Ecuyer and Debien in 1984, coronectomy has since gained popularity as a safer option in high-risk cases where the third molar roots are in close proximity to the inferior alveolar canal [12]. Also known as partial odontectomy, the procedure consists of removing the crown of the third molar while intentionally leaving the roots in place to prevent direct trauma to the inferior alveolar nerve [6,13], sectioning the tooth at the cemento-enamel junction, removing the crown, and smoothing the root surface to prevent sharp edges that could cause later complications. Unlike conventional extraction, the pulp is typically left untreated, as studies suggest it undergoes aseptic necrosis without leading to major clinical issues [6,14]. Other than being contraindicated in cases where the tooth is non-vital, has periapical pathology, extensive caries, or mobile roots [9,14,15,16], coronectomy is generally preferred in cases where radiographic signs suggest a high risk of nerve injury, such as root darkening, loss of lamina dura, or deviation of the inferior alveolar canal, which can be identified using panoramic radiographs and cone-beam computed tomography (CBCT) [5,7,8,17].

However, despite its advantages, coronectomy remains controversial among oral and maxillofacial surgeons [8]. Nonetheless, complications after coronectomy, although occurring at a lower rate compared to complete extraction, comprise nerve injuries, infection (2.49%), alveolar osteitis (1.22%), and post-operative pain (9.55%) [12,15,18].

Additional concerns include the risk of infection in retained roots and their long-term stability [8,19]. Indeed, although most retained roots remain asymptomatic, root migration, typically stabilizing within one year postoperatively [15], has been reported in 13% to 85% of cases, eventually requiring secondary surgical intervention [3,14,18] due to root exposure, infection, or patient discomfort [11,20].

Therefore, considering that the need for reintervention is one of the primary concerns associated with coronectomy [3] and the absence of prior umbrella reviews on the topic, the present systematic review of systematic reviews was conducted for two reasons: first to estimate reintervention rate and timing following coronectomy of the mandibular third molar, and second, to evaluate these outcomes in relation to the indications for reintervention, as well as patient, tooth, and coronectomy characteristics.

## 2. Materials and Methods

### 2.1. Study Protocol

The study protocol was developed according to the PRISMA (Preferred Reporting Items for Systematic Reviews and Meta-analyses) 2020 statement [21,22,23] and was registered on PROSPERO (CRD4202455688).

The formulation of the study question, the definition of the search strategies, and the criteria for study selection were developed according to the PICO model [24]. The study question [25] was “What are the rate and timing of reintervention following coronectomy of the mandibular third molar, and how are these outcomes related to the indications for reintervention, as well as sample, tooth, and coronectomy characteristics?”, focusing on:

- Population (P): subjects who have undergone coronectomy at least 6 months prior to re-intervention;
- Intervention (I): re-intervention following coronectomy;
- Comparison (C): subgroup analyses according to different indications for re-interventions, and sample, tooth, and coronectomy characteristics;
- Outcome(s) (O): re-intervention rate and timing.

### 2.2. Search Strategy

Systematic reviews with or without meta-analysis published in English without date restriction were electronically searched across databases, including Scopus, MEDLINE/PubMed, BioMed Central, Web of Science, Cochrane Library, and the PROSPERO International Prospective Register of Systematic Reviews, until 30 December 2024.

The search strategy combined medical subject headings (MeSH) and non-MeSH terms using Boolean operators, as follows: (coronectomy OR “partial odontectomy” OR “intentional root retention” OR “root retention technique”) AND (“third molar” OR “inferior molar” OR “wisdom tooth” OR “wisdom teeth”).

The following filters were applied: “Article title, Abstract, Keywords; Document type: Review; Language: English” in the Scopus database; “Article Type: Systematic Review; Article Language: English” in the MEDLINE/PubMed database; No filters in Cochrane Library; Document type: Review; Language: English” in the Web of Science database; “Published” filters (quotation marks were removed from the search string) in the PROSPERO register.

The full search strategy for each database is displayed in Supplementary File S1.

### 2.3. Study Selection and Eligibility Criteria

The collected citations were documented, and duplicates were removed using the Mendeley Reference Manager. The remaining titles were screened by two independent reviewers (F.D.S. and I.R.). These reviewers also independently screened the potentially relevant title–abstracts of systematic reviews, whether they included meta-analyses or not. Full texts of the records that met the eligibility criteria and those with ambiguous title–abstracts were obtained. Since all full texts were available, contacting the study authors was unnecessary. The full texts were independently reviewed by two authors (F.D.S. and I.R.). Any disagreements were resolved through discussion and consensus with a third author (M.A.) when necessary.

Inclusion Criteria:

- Subjects undergoing coronectomy
- Follow-up period ≥ 6 months [11,26].

Exclusion Criteria:

- Non-vital third molars, caries, endodontic disease [27]
- Wisdom teeth associated with apical pathology or apical cystic/neoplastic lesions [27]
- Systematic reviews (with or without meta-analysis), including studies where coronectomy was performed on teeth other than the lower third molar

The same study selection process was conducted on the reference lists of the articles presently included.

### 2.4. Data Extraction and Collection

Data extraction was carried out independently by two authors (F.D.S. and I.R.), utilizing a standardized form designed based on the models suggested for intervention reviews of RCTs and non-RCTs [25]. In instances of disagreement, a third author (M.A.) helped the other two to reach a consensus.

For each systematic review, data across the following main domains were extracted: studies; population, inferior third molar characteristics [28]; coronectomy; re-intervention rate; timing and collection.

A detailed description of extracted variables for each of the main domains is provided in Supplementary File S2.

### 2.5. Data Synthesis

Data were synthesized narratively, focusing on the population, intervention, comparison, and outcomes. Statistical analysis was conducted to determine the re-intervention rates and indications, with descriptive statistics used to summarize secondary outcomes. Microsoft Excel was used for data management and analysis (Microsoft Corporation, Redmond, WA, USA):

▪ to estimate re-intervention rate and timing following coronectomy of the mandibular third molars;
▪ to characterize re-intervention indications, pharmacological treatments, and patient-related outcomes;
▪ to assess re-intervention rate, timing in relation to various indications for re-intervention, and sample (size, gender ratio, and mean age, comorbidities, ongoing pharmacological treatments), third molar treated (total number, side, root morphology, angulation, distal space, depth, proximity to anatomical structures, degree of impaction—erupted/semierupted/partial bony impaction/intraosseous, presence/absence of the second molar), and coronectomy (pre-operative radiography, indications, post-operative pharmacological treatment(s), complications, follow-up (months), failure rate (tooth extracted) [14], and patient-related outcomes) characteristics.

### 2.6. Quality Assessment and Overlap Management

The quality assessment of included systematic reviews was judged using the AMSTAR 2 (A Measurement Tool to Assess Systematic Reviews) [29] accessed online on 20 January 2025 (https://amstar.ca). Two authors independently judged the included systematic review (F.D.S. and I.R.), and any discrepancies were resolved with a third author (M.A.).

As recommended by AMSTAR 2 [29], critical domains were identified to determine overall confidence ratings. The critical domains included those related to protocol registration, search strategy, justification for the excluded studies, risk of bias assessment and interpretation, and methods used for data synthesis and interpretation [29]. Based on the judgment in these critical domains, each systematic review was rated as high, moderate, low, or critically low quality, following the AMSTAR 2 overall confidence rating [29].

The overall methodological quality informed the interpretation of findings: greater weight was given to conclusions from reviews with high or moderate confidence ratings, while results from reviews rated as low or critically low were interpreted with caution and highlighted as methodologically limited.

The overlap management was assessed to evaluate the degree of overlap of the primary studies included in each systematic review, by calculating the Corrected Cover Area (CCA), as proposed by Pieper et al. [30]. The degree of the overlap was judged as “slight” (0–5%); “moderate” (6–10%); “high” (11–15%), or “very high” (>15%).

## 3. Results

### 3.1. Study Selection

The electronic search yielded 83 records in total, in particular, 21 from MEDLINE/PubMed, 31 from Scopus, 21 from Web of Science, eight from BioMed Central databases, one from the Cochrane Library, and one from the PROSPERO registry. Before the screening, 33 duplicate records were removed.

The remaining 50 records were screened and title–abstracts; after this process, 32 records did not meet the eligibility criteria and were therefore excluded.

Of the remaining 18 reports, the full-texts were read. An additional 12 reports were excluded because they did not meet the study’s inclusion/exclusion criteria. Specifically: eight studies did not report the re-intervention rate after coronectomy; two were not systematic reviews; one study reported coronectomy performed for the upper third molar; one performed a follow-up < 6 months.

In total, six systematic reviews [3,8,12,13,14,20] were included through the electronic search in the present umbrella review.

The same study selection process was carried out by manually reviewing the reference lists of the included systematic reviews.

From the reference lists of the studies included via databases and registers, a total of 310 records were identified. Before the screening, 137 duplicate records were removed.

The remaining 173 records were screened, and title–abstracts were read, after which 163 were found to not meet the eligibility criteria and were therefore excluded.

Of the remaining 10 reports, the full-texts were read, and all of them were excluded because they did not meet the study’s inclusion/exclusion criteria. Specifically: three studies did not report the re-intervention rate after coronectomy, and seven were not systematic reviews.

No additional articles were included from the manual search in the present umbrella review, which included six systematic reviews in total [3,8,12,13,14,20].

It was not necessary to contact the study authors to obtain the full-text of articles, and no further information was required during the study selection process.

**Figure 1:**
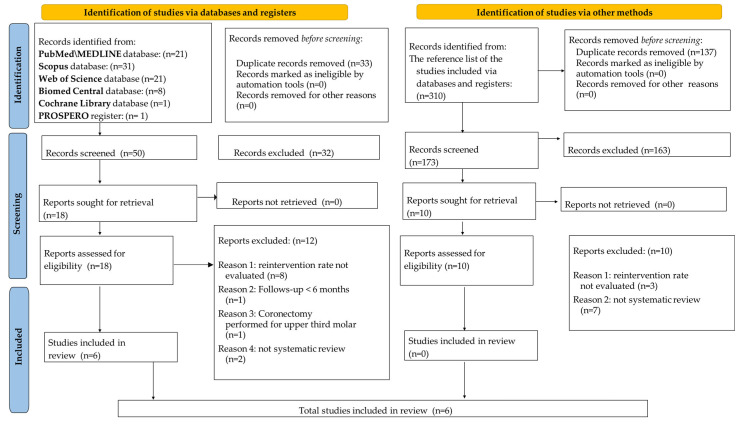
PRISMA 2020 flow-diagram study selection through electronic and manual searching.

Figure 1 shows the PRISMA flow-diagram study selection, which included electronic searching via databases and registries, and manual searching via other methods.

Data from six systematic reviews [3,8,12,13,14,20] reporting re-intervention indications and rates after at least 6 months from the coronectomy of the mandibular third molar were extracted and synthesized. Data concerning third mandibular molars that were surgically extracted were not collected.

### 3.2. Study Characteristics

A total of six systematic reviews [3,8,12,13,14,20] were included; three of them had a meta-analysis [3,12,13], and three did not [8,14,20]. In total, the six reviews examined 81 studies: eight randomized controlled trials (RCTs); 29 cohort, 22 case-control, seven prospective, and two retrospective studies. The remaining 15 studies did not define their design.

Table 1 synthesizes exclusively data extracted and collected from the included systematic reviews [3,8,12,13,14,20] reporting outcomes registered after at least 6 months from coronectomy of the mandibular third molar.

**Table 1:** Data extracted and collected from the included studies reporting outcomes registered after at least 6 months from coronectomy of the mandibular third molar.

Studies	Population Characteristics	Inferior Third Molar Characteristics	Coronectomy	Re-Intervention Rate, Timing, Indications
Barcellos B.M., 2019 J Oral Maxillofac Surg [20] Studies: *n.*15 Studies Design: N/D Moderate quality No Meta-analysis No funding	Sample size: *n.*1664 Mean age/range: N/D *n.*48 (41.4%) subjects ≤29 y.o.; *n.*42 (36.2%) subjects between 30–39 y.o.; *n.*26 (22.4%) subjects with ≥40 y.o.	Side (L/R): MD Root morphology: MD Distal space: MD Depth: MD Angulation: MD Proximity to anatomical structures: MD Degree of impaction: MD Absence of the second molar: MD	*n.*: 2062 Pre-operative radiography: MD Indications: MD Post-operative pharmacological treatment(s): MD Complications: IANI: MD t-IANI: MD p-IANI: MD LNI: MD Pain: N/D Infections: N/D Alveolar osteitis: MD Root migration: N/D/NA Root exposure: N/D/NA Root migration (mm/timing): MD Root exposure (mm/timing): MD Follow-up (months): MD Failure (tooth extracted): MD Patient-related outcomes: MD	Re-intervention rate: *n.*105 (5.1%) Re-intervention timing: mean 10.4 months (range from 6 months to 10 years) Re-intervention indications: root migration/exposure *n* = 56 (53.33%) infection *n* = 11 (10.47%) pain *n* = 10 (9.52%) residual enamel *n* = 10 (9.52%) palpable root *n.*8 (7.62%) incomplete healing *n.*2 (1.91%) periodontal disease *n.*1 (0.95%) orthodontic procedure *n.*1 (0.95%) hyperplasia distally to second molar *n.*1 (0.95%) N/D *n.*4 (3.81%) Pharmacological Treatments after Re-intervention: MD Complications: MD Patient-related outcomes: MD
	Gender ratio: 630M/968F/66MD Comorbidities: MD Ongoing Pharmacological Treatments: MD			
Long H., 2012 J Dent Res [13] Studies: *n.*4 Studies Design: *n.*2 RCT; *n.*2 CCT Critically Low Meta-analysis No funding	Sample size: *n.*460 Mean age/range: MD Gender ratio: MD Comorbidities: MD Ongoing Pharmacological Treatments: MD	Side (L/R): MD Root morphology: MD Distal space: MD Depth: MD Angulation: MD Proximity to anatomical structures: MD Degree of impaction: MD Absence of the second molar: MD	*n.*: 401 Pre-operative radiography: MD Indications: MD Post-operative pharmacological treatment(s): MD Complications: IANI: *n.*2 (0.50%) t-IANI: MD p-IANI: MD LNI: MD Pain: *n.*93 (23.19%) after 1 week Infections: *n.*14 (3.49%) Alveolar osteitis: *n.*9 (2.24%) Root migration: *n.*191 (47.63%)/NA Root exposure: MD/NA Root migration (mm/timing): 3.06 ± 1.67 mm/24 months 2.00 mm/13 months Root exposure (mm/timing): MD Follow-up (months): 10.6–25 months Failure (tooth extracted): *n.*59 (14.71%) Patient-related outcomes: MD	Re-intervention rate: *n.*8 (2.35%) Re-intervention timing: MD Re-intervention indications: Patient’s request: *n.*1 Root exposure: *n.*2 MD: *n.*5 Pharmacological Treatments after Re-intervention: MD Complications: MD Patient-related outcomes: MD
Mann A., 2021 Aust Dent J [8] Studies: *n.*6 Studies Design: *n.*3 CCT; *n.*2 RCT; *n.*1 PCS Moderate No Meta-analysis No funding	Sample size: *n.*N/D Mean age/range: MD Gender ratio: MD Comorbidities: MD Ongoing Pharmacological Treatments: MD	Side (L/R): MD Root morphology: MD Distal space: MD Depth: MD Angulation: MD Proximity to anatomical structures: MD Degree of impaction: MD Absence of the second molar: MD	*n.*: 544 Pre-operative radiography: *n.* N/D computed tomography; *n.*N/D cone beam computed tomography; *n.*N/D panoramic radiograph Indications: MD Post-operative pharmacological treatment(s): MD Complications: IANI: *n.*2 (0.37%) t-IANI: MD p-IANI: MD LNI: *n.*0 (0%) Pain: *n.*93 (17.10%) Infections: *n.*14 (2.57%) Alveolar osteitis: *n.*10 (1.44%) Root migration: *n.*238 (43.75%)/NA Root exposure: *n.*13 (2.39%)/NA Root migration (mm/timing): MD Root exposure (mm/timing): MD Follow-up (months): 6–25 months Failure (tooth extracted): *n.*68 (12.5%) Patient-related outcomes: MD	Re-intervention rate: *n.*9 (1.89%) Re-intervention timing: MD Re-intervention indications: MD Pharmacological Treatments after Re-intervention: MD Complications: MD Patient-related outcomes: MD
Martin A., 2015 Head Face Med [14] Studies: *n.*10 Studies Design: *n.*4 PCS; *n.*2 RCT; *n.*2 CCT; *n.*2 RS Moderate No Meta-analysis No funding	Sample size: *n.*789 Mean age/range: range 27.2–41.3 y.o. Gender ratio: 256M/328F/205MD Comorbidities: *n.*789 none Ongoing Pharmacological Treatments: MD	Side (L/R): MD Root morphology: MD Distal space: MD Depth: MD Angulation: MD Proximity to anatomical structures: *n.*832 to IAN Degree of impaction: MD Absence of the second molar: MD	*n.*: 832 Pre-operative radiography: *n.*N/D cone beam computed tomography *n.*N/D panoramic radiograph Indications: MD Post-operative pharmacological treatment(s): *n.*171 paracetamol and codein for 3 days *n.*163 antibiotics *n.*88 antibiotics, benzydamine hydrochloric acid, and CHX gluconate for 5 days *n.*43 antibiotics for 4 days, ibuprofen and CHX for 10 days *n.*94 pre-operative CHX mouth washes *n.*50 pre-operative antibiotics Complications: IANI: *n.*13 (1.56%) t-IANI: *n.*11 (1.32%) p-IANI: *n.*2 (0.24%) LNI (t-LNI): *n.*2 (0.24%) Pain: *n.*98 (11.78%) Infections: *n.*19 (2.28%) Alveolar osteitis: *n.*22 (2.64%) Pulp disease: *n.*1 (0.12%) Root migration: *n.*251 (30.17%)/NA Root exposure: MD/NA Root migration (mm/timing): range 1.6–1.9 mm/3 months range 2–3.4 mm/6 months range 2–3.8 mm/12 months range 3.1–4 mm/24 months Root exposure (mm/timing): MD Follow-up (months): 9.3–40 months Failure (tooth extracted): *n.*61 (7.33%) Patient-related outcomes: MD	Re-intervention rate: *n.*23 (2.98%) Re-intervention timing: MD Re-intervention indications: MD Pharmacological Treatments after Re-intervention: MD Complications: MD Patient-related outcomes: MD
Peixoto A.O., 2024 J Oral Maxillofac Surg [12] Studies: *n.*42 Studies Design: *n.*29 cohort studies; *n.*13 CCT Critically Low Meta-analysis No funding	Sample size: *n.*2983 Mean age/range: 28 ± 6.93 (range: 12–95) y.o. Gender ratio: 806M/1352F/825MD Comorbidities: *n.*2 diabetes type I Ongoing Pharmacological Treatments: MD	Side (L/R): MD Root morphology: MD Distal space: MD Depth: MD Angulation: MD Proximity to anatomical structures: *n.*3904 to IAN Degree of impaction: MD Absence of the second molar: MD	*n.*: 3904 Pre-operative radiography: *n.*N/D panoramic radiographic *n.*N/D cone beam computed tomographic *n.*N/D computed tomographic *n.*N/D spiral tomographic *n.*N/D intra oral periapical radiographic Indications: MD Post-operative pharmacological treatment(s): MD Complications: IANI: *n.*39 (0.99%) t-IANI: MD p-IANI: MD LNI: *n.*5 (0.12%) Pain: *n* = 406 (10.39%) Infections: *n* = 143 (3.66%) Alveolar osteitis: *n.*46 (1.17%) Root migration: *n.*312 (7.99%)/NA Root exposure: MD/NA Root migration (mm/timing): 2.83 ± 1.50 (range 0–6.68) mm/17.3 months Root exposure (mm/timing): MD Follow-up (months): 23.4 ± 24.05 Failure (tooth extracted): *n.*97 (2.48%) Patient-related outcomes: MD	Re-intervention rate: *n.*200 (5.25%) Re-intervention timing: MD Re-intervention indications: MD Pharmacological Treatments after Re-intervention: MD Complications: MD Patient-related outcomes: MD
Pitros P., 2020 Br J Oral Maxillofac Surg [3] Studies: *n.*4 Studies Design: *n.*2 RCT; *n.*2 CCT Low Meta-analysis No funding	Sample size: MD Mean age/range: MD Gender ratio: MD Comorbidities: MD Ongoing Pharmacological Treatments: MD	Side (L/R): MD Root morphology: MD Distal space: MD Depth: MD Angulation: MD Proximity to anatomical structures: *n.*455 to IAN Degree of impaction: MD Absence of the second molar: MD	*n.*: 455 Pre-operative radiography: MD Indications: MD Post-operative pharmacological treatment(s): MD Complications: IANI: *n.*6 (1.32%) t-IANI: MD p-IANI: MD LNI: MD Pain: *n.*93 (20.44%) Infections: *n.*14 (3.08%) Alveolar osteitis: *n.*13 (2.86%) Root migration: N/D/NA Root exposure: N/D/NA Root migration (mm/timing): MD Root exposure (mm/timing): MD Follow-up (months): mean 16.5 months Failure (tooth extracted): MD Patient-related outcomes: MD	Re-intervention rate: *n.*7 (1.54%) Re-intervention timing: MD Re-intervention indications: infections: *n.*5 root exposure: *n.*1 patient’s request: *n.*1 Pharmacological Treatments after Re-intervention: MD Complications: MD Patient-related outcomes: MD

Abbreviations: number, “n.”; Male, “M”; Female, “F”; years old, “y.o.”; percentage, “%”; millimeters, “mm”; Prospective Cohort studies, “PCS”; Prospective Study, “PS”; Retrospective Study, “RS”; Case-Control Trial, “CCT”; Randomized Controlled Trials, “RCT”; Missing Data, “MD”; Not Defined, “N/D”; Not Applicable, “NA”; chlorhexidine, “CHX”; inferior alveolar nerve, “IAN”; inferior alveolar nerve injury, “IANI”; transitory-IANI, “t-IANI”; permanent-IANI, “p-IANI”; lingual nerve injury, “LNI”.

The study population was reported by four [12,13,14,20] of the six systematic reviews included. In total, 5896 subjects were included, ranging from 12 to 95 years old [12,14,20]. Three studies [12,14,20] specified the gender ratio (1692M/2648F), which was M:F = 1:1.57. In one study [20] two subjects had type 1 diabetes, while one study [14] declared that their study population did not have any comorbidities (*n* = 789 subjects). No study reported the ongoing pharmacological treatments of study participants.

Three studies [3,12,20] recorded the anatomical proximity of teeth treated with coronectomies with the inferior alveolar nerve (IAN), for a total of 5191 teeth.

No study reported data regarding the site (left/right) of the third mandibular molar, root morphology, distal space, depth, angulation, degree of impaction, or the absence of the second mandibular molar.

The overall number of coronectomies performed was 8198 [3,8,12,13,14,20] of which 7913 were successful, not requiring immediate tooth extractions. The pre-operative radiography used to evaluate the anatomical relationship of third mandibular molars with the other structures was: panoramic radiograph [8,12,14], computed tomography [8,12], cone beam computed tomography [8,12,14], spiral tomography [12], and intra-oral periapical radiograph [12]. One study [14] reported the pharmacological treatment prescribed after coronectomy, in particular: in 171 cases, paracetamol and codeine for 3 days; in 163, antibiotics; in 88, antibiotics plus benzydamine hydrochloric acid and CHX gluconate for 5 days; in 43, antibiotics for 4 days plus ibuprofen and CHX for 10 days; in 94, pre-operative CHX mouthwashes; and in 50, pre-operative antibiotics were prescribed. The timing of follow-ups after coronectomy ranged from 6 months [8] to 40 months [14].

### 3.3. Re-Intervention Outcomes (Rate, Timing, Indications)

Re-intervention after at least 6 months was necessary in 352 cases (re-intervention rate = 4.45%).

The timing of re-intervention after coronectomy was recorded by one study [20], ranging from 6 months to 10 years, with a mean of 10.4 months.

**Figure 2:**
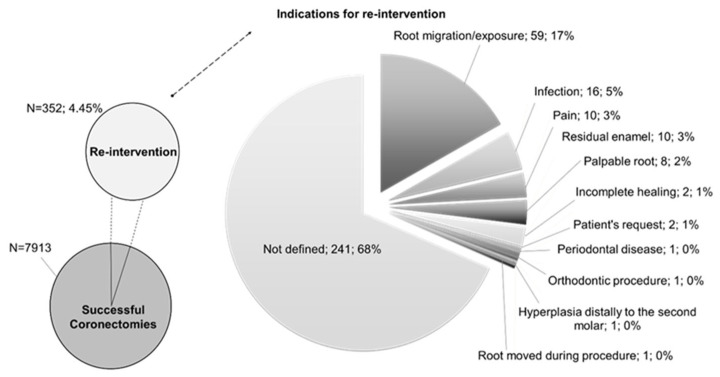
Pie chart of re-intervention rate and indications.

The indications for re-intervention (Figure 2) were reported in three studies [3,13,20]. Specifically, the re-interventions were performed due to root exposure in 59 cases (16.76%) [3,13,20], infection in 16 cases (4.55%) [3,20], pain in 10 cases (2.84%) [20], residual enamel in 10 cases (2.84%) [20], palpable root in eight cases (2.27%) [20], incomplete healing in two cases (0.57%) [20], patient’s request in two cases (0.57%) [3,13], periodontal disease in one case (0.28%) [20], orthodontic procedure in one case (0.28%) [20], hyperplasia distally to the second molar in one case (0.28%) [20], and root moved during the procedure in one case (0.28%) [20]. In the remaining 241 cases, the indications for the re-intervention were not specified.

No study reported whether pharmacological treatment was prescribed after re-intervention, nor did they register the complications that occurred after re-intervention.

### 3.4. Other Complications and Root Migration Following Coronectomy

Five systematic reviews [3,8,12,13,14] reported the complications that occurred after coronectomies, as summarized in Table 2.

**Table 2:** Complications registered after successful coronectomies, number of cases, percentage of cases with complications of total coronectomies performed (*n* = 8198), reported range of complications in the included systematic reviews and reference(s).

Complications	N. of Complications After Coronectomies	% of Complications After Coronectomies	Reported Range of Complications	Reference(s)
IANI	62 (11 were t-IANI, 2 p-IANI, 49 N/D)	0.78%	from 0.37% [8] to 1.56% [14]	[3,8,12,13,14]
LNI	7 (2 were t-LNI, 5 N/D)	0.09%	from 0% [8] to 0.24% [14]	[8,12,14]
Pain	783	9.90%	from 10.39% [12] to 23.19% [13]	[3,8,12,13,14]
Infection	204	2.58%	from 2.28% [14] to 3.66% [12]	[3,8,12,13,14]
Alveolar osteititis	100	1.26%	from 1.44% [8] to 2.86% [3]	[3,8,12,13,14]
Root migration	992	12.54%	from 7.99% [12] to 47.63% [13]	[8,12,13,14]
Root exposure	13	0.16%	2.39% [8]	[8]
Pulp disease	1	0.01%	0.12% [14]	[14]
Overall	2162	27.32%		

Abbreviations: number, “n”; percentage, “%”; Not Defined, “N/D”; inferior alveolar nerve injury, “IANI”; transitory-IANI, “t-IANI”; permanent-IANI, “p-IANI”; lingual nerve injury, “LNI”; transitory-LNI, “t-LNI”.

The rate of root migration was registered in three studies [8,12,14] and demonstrates a progressive pattern over time, with most studies reporting a cumulative displacement ranging from approximately 2 mm to 4 mm within the first 24 months (Table 3). Martin et al. (2015) [14] documented incremental migration from 3 to 24 months, whereas Long et al. (2012) [13] and Peixoto et al. (2024) [12] provided mean values with standard deviations, confirming interindividual variability. Peixoto et al. reported the widest range of displacement (0–6.68 mm) at a mean follow-up of 17.3 months.

**Table 3:** Rate of root migration following coronectomy over time.

Follow-Up Interval	Mean ± SD (mm)	Range (mm)	Study (Year)
3 months	—	1.6–1.9	Martin et al. (2015) [14]
6 months	—	2.0–3.4	Martin et al. (2015) [14]
12 months	—	2.0–3.8	Martin et al. (2015) [14]
13 months	2.00	—	Long et al. (2012) [13]
17.3 months	2.83 ± 1.50	0–6.68	Peixoto et al. (2024) [12]
24 months	3.06 ± 1.67	3.1–4.0	Long et al. (2012) [13]

### 3.5. Quality Assessment and Overlap Management

Three systematic reviews [8,14,20] were judged as moderate quality, two [12,13] as critically low quality, and one [3] as low quality, using the AMSTAR 2 tool [29] (Table 4).

**Table 4:** Quality assessment of the included systematic reviews using the AMSTAR 2 tool.

		Barcellos B.M., 2019, [20]	Long H., 2012, [13]	Mann A., 2021, [8]	Martin, A., 2015, [14]	Peixoto A.O., 2024, [12]	Pitros P., 2020, [3]
Item 1		Y	Y	N	Y	Y	Y
Item 2 *		PY	PY	PY	Y	Y	Y
Item 3		Y	Y	Y	Y	Y	Y
Item 4 *		Y	N	PY	Y	PY	Y
Item 5		Y	N	N	N	Y	N
Item 6		Y	N	N	N	Y	N
Item 7 *		Y	Y	Y	Y	N	Y
Item 8		N	Y	PY	Y	PY	N
Item 9 *	9.a	PY	N	Y	Y	Y	N
	9.b	PY	N	Y	Y	Y	N
Item 10		NA	N	NA	N	N	N
Item 11 *	11.a	NA	Y	NA	NA	Y	Y
	11.b	NA	Y	NA	NA	Y	Y
Item 12		NA	Y	NA	NA	N	Y
Item 13 *		Y	Y	Y	Y	Y	N
Item 14		N	Y	Y	Y	Y	N
Item 15 *		NA	Y	NA	NA	N	Y
Item 16		N	N	Y	N	N	Y
Quality		Moderate	Critically Low	Moderate	Moderate	Critically Low	Low

Abbreviations: Yes, “Y”; No, “N”; Partial Yes, “PY”; Not Applicable, “NA”; AMSTAR 2 critical items, “*”.

The overlap management was assessed to evaluate the degree of overlap of the primary studies included in each systematic review by calculating the CCA, as proposed by Pieper et al. [30]; the degree of overlap was 18%, which was judged as “very high”.

## 4. Discussion

Coronectomy carries a small risk of failure rates and complications, representing a safer alternative for high-risk third molar extractions, particularly concerning nerve preservation.

Regarding coronectomy failure rates, the present study’s 3.48% overall failure rate (ranging from 2.48% to 14.71%) [8,12,13,14] is within the spectrum reported in the literature. Simons et al. (2024) [31] analyzed 813 coronectomy cases and found failure rates ranging from 2% to 15%, with higher failure rates observed in Pell and Gregory Class III, Position B cases. Similarly, Nowak et al. (2024) [32] reported an overall coronectomy success rate of 93%, with intraoperative failure occurring in 3.6% of cases [32]. Their study further identified pain (15%), infection (9%), and dry socket (3.6%) as the most common postoperative complications. Reoperation was required in 1.8% of cases due to root migration or infection.

Pang et al. (2024) [33] reported coronectomy success rates of 93%, with failure rates ranging from 3.4% to 6.8%, reinforcing that coronectomy is a relatively safe procedure with a moderate risk of complications and failure. Indeed, a systematic review by Kostares et al. (2024) [34] examined postoperative infection rates following coronectomy, reporting a pooled surgical site infection prevalence of 2.4% [34]. Consistently, Hamad et al. (2024) [35] demonstrated that coronectomy is a viable alternative to complete extraction in cases with high IAN proximity. Their findings showed that the risk of inferior alveolar nerve injury was significantly lower in the coronectomy group (0.5%) compared to the total extraction group (3.7%) [35]. Furthermore, the likelihood of root migration was found to increase over time, with 74% of retained roots exhibiting migration at 12 months postoperatively, though only a small percentage required secondary intervention.

However, coronectomy is often avoided due to concerns regarding root infection, the long-term stability of retained roots, and the need for secondary interventions [8,19], whose rate, timing, and indications have been presently assessed.

### 4.1. Reintervention Outcomes (Rate, Timing, Indications, Complications)

#### 4.1.1. Reintervention Rate

The necessity for re-intervention following coronectomy in the present study was observed in 4.29% of cases, ranging from 1.54% to 5.12%, with retained roots being the primary reason for secondary procedures in most cases [3,12,36]. These findings align with Póvoa et al. (2021) [37], who reported a lower re-intervention rate of 1.13%, suggesting that differences in clinical protocols and patient selection criteria may influence the likelihood of requiring secondary interventions. Similarly, Nowak et al. (2024) [32] reported a re-intervention rate of 3.1%, with the majority of cases attributed to root migration leading to exposure or symptomatic root retention. Their study found that reoperation was more common in younger patients and those with pre-existing periodontal issues, reinforcing the importance of patient selection in coronectomy planning.

Additionally, Bailey et al. (2020) [38] highlighted variability in coronectomy out-comes, noting that re-intervention rates can be influenced by root migration patterns, surgical techniques, and patient-specific anatomical factors. Their systematic review indicated that successful coronectomy cases generally exhibit minimal root movement and a stable bone healing process. These findings are further corroborated by Hamad et al. (2024) [35], who found that root migration was significantly correlated with coronectomy failure, with 5.6% of cases requiring secondary extraction due to root exposure or persistent infection [36].

The present study’s re-intervention rate falls within the range reported in the literature, confirming that while coronectomy is a viable option for reducing inferior alveolar nerve injury, monitoring for root migration and potential complications is essential. Ali et al. (2017) [39] emphasized the need for long-term follow-up in coronectomy patients, as delayed root migration beyond 12 months postoperatively was associated with a higher likelihood of requiring secondary intervention [28]. Additionally, Kostares et al. (2024) [34] identified surgical site infection as a significant predictor of reoperation, noting that cases with post-coronectomy infections had a 3.2-fold higher risk of requiring secondary treatment [33].

#### 4.1.2. Reintervention Timing

The timing of re-intervention was recorded by a single study, which reported that re-interventions occurred between six months and ten years post-coronectomy, with a mean time of 10.4 months [20], thus underscoring the need for long-term follow-up in coronectomy cases. Bernabeu-Mira et al. (2024) [40] reported that late root exposure and infection were primary causes for re-intervention within a 2–9 year follow-up period, suggesting that while most retained roots remain asymptomatic, a small percentage may require extraction over time.

Similarly, Nowak et al. (2024) [32] identified an average time to re-intervention of 12.2 months, with 80% of secondary extractions occurring within the first two years post-coronectomy [27]. Their study also noted that delayed root migration beyond 12 months was the primary predictor of late-stage re-intervention, reinforcing the necessity of long-term monitoring.

Similarly, Póvoa et al. (2021) [37] reported that 1.13% of cases required re-intervention, with the necessity often arising beyond the initial post-surgical period. This lower rate compared to the present study might be attributed to differences in patient selection criteria and surgical techniques. Their review also emphasized the lack of standardized follow-up protocols, which may contribute to the variability in reported re-intervention rates. These findings are further supported by Hamad et al. (2024) [35], who reported that re-intervention rates were highly dependent on follow-up duration, with 6.8% of cases requiring extraction beyond two years postoperatively.

Additionally, Kostares et al. (2024) [34] highlighted that re-intervention rates are often underestimated due to inconsistent long-term follow-up, as many studies fail to monitor patients beyond the first postoperative year. Their systematic review suggested that standardized long-term follow-up protocols should be implemented to improve the accuracy of coronectomy outcome assessments [34].

Overall, the timing of re-intervention, which has rarely been reported, aligns with previous studies, confirming that re-intervention is generally rare but necessary in a subset of cases due to root exposure or infection. However, the lack of data on post-re-intervention complications remains a significant limitation. Future research should prioritize longer follow-up durations and comprehensive reporting of secondary procedures to enhance clinical decision-making and optimize patient outcomes.

#### 4.1.3. Reintervention Indications

The present study identified root exposure (16.76%) as the most frequent reason for re-intervention, followed by infection (4.55%), pain (2.84%), residual enamel (2.84%), and other less common indications, such as palpable root (2.27%), and incomplete healing (0.57%) [3,13,20]. These findings are consistent with Agbaje et al. (2015) [36] who reported root exposure as a common post-coronectomy complication, frequently necessitating secondary intervention. Their study found that periapical infections and persistent root mobility were key indicators for re-operation, aligning with the present study’s findings.

Moreover, Nowak et al. (2024) [32] confirmed that root migration is a frequent but generally non-threatening phenomenon, with most roots stabilizing over time. However, they identified cases where excessive root migration resulted in secondary infection and necessitated extraction, particularly in high-risk patients. Their findings suggest that root migration should be closely monitored, as unpredictable movement patterns may contribute to long-term complications.

Similarly, Póvoa et al. (2021) [37] identified root migration (also without exposure) and infection as frequent post-coronectomy complications, with re-intervention rates of 1.13%, notably lower than the rates observed in the present study. The variation may stem from differences in surgical protocols, patient selection criteria, and follow-up durations. Hamad et al. (2024) [35] further corroborated these findings, reporting that late root exposure occurred in 5.4% of cases, often due to progressive resorption of surrounding alveolar bone, emphasizing the importance of long-term radiographic monitoring to detect early signs of exposure.

Additional rare indications for re-intervention in the present study included periodontal disease (0.28%), orthodontic procedure necessity (0.28%), hyperplasia distal to the second molar (0.28%), and intraoperative root displacement (0.28%). These factors are not widely reported in the literature, highlighting a potential gap in research on long-term coronectomy outcomes. Leizerovitz & Leizerovitz (2013) [41,42] described a modified coronectomy technique involving bone grafting to mitigate the risk of periodontal complications, suggesting that adjunctive techniques may help prevent re-intervention in high-risk cases.

Moreover, patient-requested re-interventions accounted for 0.57% of cases, which is consistent with previous reports indicating patient preference as a factor influencing secondary surgical decisions. However, no study, including the present one, documented whether pharmacological treatments were prescribed post-re-intervention, highlighting an additional knowledge gap. Kostares et al. (2024) [34] emphasized that the lack of standardized post-surgical infection management contributes to variability in re-intervention rates. Their systematic review suggested that antibiotic prophylaxis and antiseptic rinses may reduce infection rates [43,44] but remain inconsistently applied across studies.

Notably, in 241 cases, the reasons for re-intervention remained unspecified. This lack of documentation reflects a broader issue in the literature, where detailed postoperative assessments and long-term monitoring strategies are often inconsistently reported. Future studies should aim to clarify secondary intervention triggers, particularly concerning less common complications.

Overall, the findings confirm that root exposure and infection are primary reasons for re-intervention, aligning with previous studies. However, the lack of data on post-re-intervention management and pharmacological treatments remains a significant limitation. Ali et al. (2017) [39] highlighted the need for standardized follow-up guidelines, advocating for routine radiographic monitoring at 6, 12, and 24 months post-coronectomy to identify early signs of complications before re-intervention becomes necessary. Future research should focus on standardized criteria for re-intervention and comprehensive reporting of secondary procedure outcomes to enhance clinical decision-making and optimize patient care.

#### 4.1.4. Other Complications and Root Migration Following Coronectomy

The complication rate observed in the present study (26.37%) aligns with previous findings, particularly regarding root migration (12.20%), the most frequent issue. Bernabeu-Mira et al. (2024) [40] and Póvoa et al. (2021) [37] reported that root migration is a common but generally non-threatening consequence, as roots tend to migrate away from the inferior alveolar nerve (IAN) and stabilize over time. However, in some cases, migration led to root exposure (0.16%), necessitating extraction, a trend similarly documented in the literature. Simons et al. (2023) [31] conducted a prospective cohort study assessing early root migration post-coronectomy, finding that the majority of root displacement occurs within the first six months, with a mean migration of 3.30 mm at two months and 5.27 mm at six months. Their findings highlight that the extent of root migration is significantly influenced by patient age and sex, with younger individuals and women experiencing greater migration rates.

Hamad et al. (2024) [35] found that root migration occurred in 74% of cases within the first 12 months postoperatively, with an average displacement of 3.85 mm, though only a small percentage required secondary intervention [35]. Similarly, Ali et al. (2017) [39] reported that root migration was most pronounced during the first postoperative year, with movement plateauing at 24 months. Their study emphasized that the risk of inferior alveolar nerve injury (IANI) during delayed root removal was minimal due to the migration of roots away from the IAN, reinforcing the long-term safety of coronectomy.

These findings confirm that while migration is common, its clinical impact is often minimal. However, as noted by Kostares et al. (2024) [34], cases where root migration results in exposure and infection may require intervention, particularly in patients with limited available distal space or unfavorable root morphology. Their systematic review suggested that careful preoperative risk assessment is crucial for identifying patients at higher risk of complications.

The systematic review by Póvoa et al. (2021) [37] indicated that root extraction after coronectomy was required in 5.28% of cases, a higher rate than observed in the present study, suggesting variability in clinical management approaches.

Pain, occurring in 9.55% of cases, is another common post-coronectomy issue. Williams and Tollervey (2016) [45] highlighted that postoperative pain following coronectomy is comparable to total extraction in the short term but tends to be less persistent due to reduced nerve trauma. However, Póvoa et al. (2021) [37] reported a higher pain rate of 22.04%, suggesting that individual factors such as surgical technique and follow-up duration may influence pain perception. Despite this discrepancy, both studies agree that coronectomy remains a viable option for minimizing long-term nerve-related pain complications. Nowak et al. (2024) [32] reported a 15% incidence of postoperative pain, with most cases resolving in the first month, further supporting that coronectomy is a relatively well-tolerated procedure with manageable discomfort levels.

Infection rates in the present study (2.49%) were lower than those reported by Póvoa et al. (2021) [37], who found an infection rate of 3.95%, likely reflecting differences in surgical protocols or patient risk factors. Yan et al. (2020) [16] similarly found that infection risk varies between 1% and 3%, depending on root positioning and pre-existing periodontal conditions. A systematic review by Kostares et al. (2024) [34] reported a pooled prevalence of surgical site infections following coronectomy at 2.4%, confirming that infection risk is generally low but influenced by procedural variables and patient-specific factors [34].

Inferior alveolar nerve injury (IANI) was documented in 0.76% of cases in the present study, aligning with Póvoa et al. (2021) [37], who reported an incidence of 0.59%. Both studies confirm that IANI following coronectomy is rare, especially compared to full extraction, which carries a significantly higher risk. Similarly, lingual nerve injury (LNI) was rare (0.09%) and consistent with the 0.22% rate reported by Póvoa et al. (2021) [37], reinforcing that atraumatic surgical techniques contribute to reducing nerve damage risk [46]. Ali et al. (2017) [42] further supported this, concluding that coronectomy significantly reduces the risk of IANI compared to total extraction, particularly in cases with high radiographic risk markers [47].

The occurrence of alveolar osteitis (1.22%) in the present study is slightly higher than the 1.12% reported by Póvoa et al. (2021) [37] but remains within the expected range for coronectomy. Bernabeu-Mira et al. (2024) [40] noted that dry socket is less frequent in coronectomy than in total extraction due to the preservation of root structures and enhanced bone healing. Additionally, Hamad et al. (2024) [35] found that dry socket was significantly lower in coronectomy cases (0.5%) compared to total extraction (3.7%), reinforcing the protective effect of coronectomy against alveolar osteitis [35].

Pulp disease (0.01%) was an exceptionally rare complication in both the present study and Póvoa et al. (2021) [37], further supporting the idea that retained roots rarely become symptomatic unless exposed to the oral cavity.

Overall, the findings of the present study are largely consistent with the literature, confirming that coronectomy effectively reduces nerve injury risks while maintaining a moderate complication rate. Root migration remains the most common issue, but it is often clinically insignificant. Infection and pain rates, while slightly variable between studies, remain within an acceptable range, and long-term complications remain rare. However, as highlighted by Kostares et al. (2024) [34], further long-term studies are needed to assess complication rates following re-intervention, particularly in cases requiring secondary extraction due to root exposure or infection.

### 4.2. Clinical Implications: Case Selection, Preoperative Imaging, Follow-Up

#### 4.2.1. Study Population Characteristics and Patient Selection

The present systematic review included a total of 5896 subjects aged between 12 and 95 years, aligning with prior research on coronectomy outcomes. In comparison, Nowak et al. (2024) [32] reported a similar age range in their cohort, with a mean age of 32 years (range: 17–91 years), Ali et al. (2017) [39] recorded a mean age of 31.7 years in a study comparing coronectomy with surgical extraction, and Bernabeu-Mira et al. (2024) [40] in a retrospective study with 2–9 years of follow-up reported a mean age of 36 years (range: 22–77 years) reinforcing the consistency of demographic trends across studies.

Gender distribution remained underreported, with only a few studies, including the current one, explicitly documenting a male–female ratio of 1:1.57 (1692 men and 2648 women) [11,45,46,47,48]. This is consistent with the Ali et al. (2017) [39] findings, who observed a comparable gender ratio in their cohort. Nowak et al. (2024) [32] also reported a slightly higher proportion of females, with 94% having no relevant medical history, while only one patient had well-controlled diabetes, and another was on low-dose systemic steroids.

Similarly, Bailey et al. (2020) [38] emphasized that many studies exclude individuals with systemic conditions, limiting the generalizability of coronectomy findings. In a systematic review on surgical site infections, Kostares et al. (2024) [34] highlighted that comorbidities such as diabetes, obesity, and lifestyle factors like smoking and alcohol consumption significantly influence postoperative outcomes.

This is consistent with the lack of documentation regarding comorbidities and pharmacological treatments in the present study, except for one study identifying two participants with Type 1 diabetes [20], and another stating that no comorbidities were present in 789 subjects [14]. However, Nowak et al. (2024) [32] found that only two patients in their cohort had documented systemic conditions, supporting the notion that most coronectomy studies underreport comorbidities.

The consistent demographic patterns observed across studies support the general applicability of coronectomy in young to middle-aged adults; however, the underreporting of comorbidities emphasizes the importance of individualized risk assessment in clinical decision-making.

#### 4.2.2. Third Molars Anatomical Characteristics, Proximity to the Inferior Alveolar Nerve, and Tooth Selection

None of the studies included reported detailed data concerning other anatomical and morphological aspects of the mandibular third molar, such as its position (left or right), root morphology, distal space, depth, angulation, degree of impaction, or the presence or absence of the second mandibular molar. This gap is particularly relevant, as Nowak et al. (2024) [32] emphasized that standardized reporting of third molar characteristics is essential in determining surgical risks and long-term outcomes. Their study found that neglecting variables such as root morphology and distal space can hinder the accurate prediction of root migration following coronectomy.

Despite the established relevance of third molar anatomical factors, especially of tooth position and root characteristics, highlighted in the study of Yan et al. (2020) [16], these variables are often not integrated into preoperative risk assessments [34], particularly regarding surgical decision-making for coronectomy versus full extraction. Accordingly, Kostares et al. (2024) [34] underscored that inadequate preoperative assessment of anatomical factors contributes to higher rates of post-surgical complications, particularly infections and prolonged healing times.

The classification of impaction types has been a subject of research, particularly in relation to the indication for coronectomy. Simons et al. (2024) [31] explored the association between mandibular third molar impaction patterns and coronectomy indications using the Pell and Gregory and Winter classification systems. Their study, which included 813 mandibular third molars from 565 patients, revealed that most impacted third molars classified as Class IIB with a mesioangular inclination were more frequently selected for coronectomy [31].

Hamad et al. (2024) [35] also demonstrated that the likelihood of root migration varies depending on the impaction depth and angulation, reinforcing the need for more comprehensive morphological assessments. Their study suggests that third molar root morphology and available distal space may affect the risk of root exposure or infection, although it does not consider left/right tooth position. However, while Simons et al.’s study [31] supports the relevance of impaction classification, it does not focus on specific anatomical factors like root morphology and distal space, which remain largely underreported in the literature.

The importance of detailed anatomical classification in predicting surgical risks and outcomes is also emphasized in the systematic review by Bailey et al. (2020) [38], which examined various surgical techniques for third molar removal. Their findings indicate that IAN injury risk is significantly influenced by the depth and angulation of impaction, reinforcing the need for standardized reporting of these variables in coronectomy studies. Compared to third molar anatomical factors, the proximity of the treated third molars to the IAN was better documented, being examined in three studies, which collectively assessed 5191 teeth [3,12,20]. This finding aligns with the consideration that the proximity of the IAN remains a primary concern when determining surgical strategies, especially in cases of impacted third molars [45].

Moreover, Agbaje et al. (2015) [36] found that impacted third molars with close proximity to the IAN had a higher incidence of root migration post-coronectomy, further reinforcing the need for precise anatomical assessments before surgery [36].

The absence of detailed reporting on key anatomical features of the mandibular third molar—such as root morphology, impaction depth, and distal space—hampers accurate surgical planning and risk stratification. Since these factors critically influence the likelihood of root migration and postoperative complications, their omission undermines the ability to appropriately select between coronectomy and extraction. Integrating standardized anatomical assessment into preoperative protocols may reduce inappropriate indications for coronectomy and, consequently, lower the observed reintervention rates.

#### 4.2.3. Preoperative Imaging

Various radiographic techniques were utilized to evaluate the anatomical relationships of mandibular third molars with surrounding structures, and the most commonly employed imaging modality was panoramic radiography [8,12,14], followed by computed tomography (CT) [8,12] and cone-beam computed tomography (CBCT) [8,12,14].

Panoramic radiography remained the most frequently used imaging modality in third molar assessment, as confirmed by Bernabeu-Mira et al. (2024) [40], who emphasized that panoramic radiographs are often the first-choice imaging tool for evaluating the proximity of third molar roots to the inferior alveolar nerve (IAN). However, the accuracy of panoramic radiography has been questioned [49] as it can misrepresent the true spatial relationship between the mandibular canal and third molar roots. Simons et al. (2024) [31] highlighted that panoramic imaging alone may not be sufficient for precise localization of the inferior alveolar nerve, necessitating further assessment using CBCT. Similarly, Nowak et al. (2024) [32] emphasized that panoramic radiography alone is insufficient for precise risk assessment, reporting that in 30% of cases, high-risk third molars classified on panoramic radiography were actually found to be at a safe distance from the IAN when assessed with CBCT.

This finding underlines the importance of three-dimensional imaging in reducing unnecessary coronectomies or complete extractions that could increase patient morbidity [32]. Accordingly, Hamad et al. (2024) [35] highlighted CBCT as the gold standard for assessing high-risk cases, particularly when three or more radiographic risk signs are detected on a panoramic radiograph. Their study demonstrated that CBCT evaluation altered the treatment plan in 26% of cases, shifting from extraction to coronectomy, and thereby reducing the incidence of IAN injury [35]. Similarly, Ali et al. (2017) [39] reinforced the CBCT role in preoperative risk stratification, proposing a “traffic light” classification system based on radiographic assessment, which demonstrated that panoramic radiographs alone were often insufficient to accurately predict nerve involvement.

These findings align with the present review, which documented the use of CBCT in selected cases [8,12,14]. Notably, Yan et al. (2020) [16] demonstrated that CBCT offers superior three-dimensional visualization of the spatial relationship between the third molar and surrounding structures, proving particularly valuable in assessing IAN injury risk prior to coronectomy. By enabling a more detailed evaluation of root morphology, positioning, and impaction depth, CBCT facilitates more precise surgical planning [16]. Supporting this, Agbaje et al. (2015) [36] found that the absence of cortication between the mandibular canal and third molar root, as detected via CBCT, was a more reliable predictor of nerve injury risk than traditional panoramic radiographic markers [36].

Further reinforcing the value of CBCT, Bernabeu-Mira et al. (2024) [40] confirmed its growing recommendation for high-risk cases, particularly where panoramic imaging alone is inadequate in determining the extent of IAN proximity [43]. Nowak et al. (2024) [32] validated these findings, demonstrating that CBCT-assisted decision-making significantly reduced unnecessary coronectomies while enhancing patient outcomes [32]. Their retrospective study underscored the crucial role of CBCT in identifying cases where coronectomy was preferable to total extraction. Additionally, Kostares et al. (2024) [34], in a systematic review, emphasized the role of CBCT in minimizing surgical site infections by enabling more precise preoperative planning, reducing unnecessary tissue trauma, and consequently lowering post-operative infection rates [34].

Additionally, spiral tomography [12] and intra-oral periapical radiography [12] were used in some cases, being less commonly utilized in third molar evaluation. Williams & Tollervey (2016) [44] noted that CT scans can provide valuable additional information, but their higher radiation dose makes them less desirable for routine use in dental practice. However, they also emphasized that CT may be justified in complex cases requiring a precise assessment of the third molar’s relationship with adjacent structures.

Intra-oral periapical radiography was used in some cases in the present study [12]. While periapical radiographs offer high resolution, Pang et al. (2024) [33] reported that they provide limited information regarding the three-dimensional orientation of impacted third molars. Their study suggested that while periapical radiographs are effective in detecting localized pathology, such as periapical lesions or caries, they are insufficient for evaluating surgical complexity in impacted third molar cases. Furthermore, Ali et al. (2017) [39] corroborated these findings, stating that periapical radiography lacks the spatial accuracy required for assessing root morphology and its relation to the mandibular canal, making CBCT a more reliable alternative in complex cases.

In light of the limitations of two-dimensional imaging, the use of CBCT plays a pivotal role in accurately identifying high-risk anatomical relationships, thereby guiding the selection between coronectomy and complete tooth extraction, beyond reducing the likelihood of inferior alveolar nerve injury, and the rate of secondary interventions.

#### 4.2.4. Follow-Up Period

The follow-up periods in the present study, ranging from six [8] to 40 months [14], align with findings from prior studies, but may not be sufficient for assessing long-term outcomes. Indeed, Bernabeu-Mira et al. (2024) [40] reported follow-ups extending up to 84 months, emphasizing that longer observation allows for better evaluation of root migration, secondary complications, and re-intervention needs.

Similarly, Póvoa et al. (2021) [37] recommended at least two years of follow-up to detect late root exposure and periodontal changes, cautioning that shorter studies might underreport delayed complications.

Also, Bailey et al. (2020) [38] noted that many studies limit follow-ups to 12–24 months, potentially missing long-term complications, such as late infections or the need for secondary extractions.

Their findings underscore the importance of standardized follow-up protocols to enhance consistency and comparability in coronectomy research. Accordingly, while the present study’s follow-up periods fall within reported ranges, extending observational timeframes beyond three years could improve the accuracy of complication detection and optimize clinical management strategies; thus, researchers should advocate for longer, standardized follow-ups to better assess the long-term success and safety of coronectomy.

### 4.3. Methodological Considerations and Limitations

The present umbrella review offers a comprehensive synthesis of available evidence on coronectomy outcomes, providing a broad perspective on patient demographics, complication rates, and re-intervention timing. It uncovers critical gaps in the literature, such as the absence of standardized imaging protocols, inconsistent follow-up durations, and limited reporting on re-intervention outcomes and long-term patient management. The inclusion of diverse study designs contributes to a more inclusive overview of current clinical practice.

However, these strengths are tempered by several limitations, discussed below.

#### 4.3.1. Study Design, Data Reporting and Integration

Although this umbrella review consolidated evidence from six systematic reviews [3,8,12,13,14,20], the evidence base comprises randomized controlled trials, prospective and retrospective cohort studies, and case-control studies, each differing in methodological rigor and susceptibility to bias, complicating the interpretation of aggregated findings and weakens the overall coherence of the umbrella review.

This challenge is further compounded by persistent gaps in clinical and anatomical data reporting. Key variables such as root morphology, impaction depth, third molar position, distal space, and presence of adjacent second molars were frequently omitted or inconsistently documented [12,13,14,20], despite their established relevance in surgical planning and postoperative risk stratification. While the proximity of the inferior alveolar nerve was more consistently reported [3,12,20], the lack of standardized anatomical classification restricted meaningful comparisons. The geopardized lack of data precluded integrating or quantificating findings across reviews.

Likewise, comorbidities, pharmacological treatments, and long-term outcomes following re-intervention were rarely reported, thereby reducing the generalizability of findings to real-world clinical populations. Moreover, inconsistencies in reporting and methodological variability hinder the development of higher-level conclusions and limit the practical translation of findings into clinical decision-making.

#### 4.3.2. Quality of Included Reviews and Potential for Publication Bias

The methodological quality of the included reviews, assessed using the AMSTAR 2 tool [26], was variable. Three reviews were rated as moderate in quality [8,14,20], while two were critically low [12,13], and one was low [3]. Deficiencies were primarily related to the absence of protocol registration, insufficient risk of bias assessments, and incomplete reporting of funding sources. These weaknesses undermine the strength of the synthesized evidence and highlight the need for greater methodological rigor in future systematic reviews on coronectomy.

Publication bias also remains a notable concern for overrepresentation of studies highlighting the safety of coronectomy or the diagnostic value of CBCT. Addressing this issue in future reviews will be essential for maintaining balanced interpretations and evidence-based clinical recommendations.

#### 4.3.3. Study Overlap and Redundancy

The CCA of 18% highlighted a very high overlap of primary studies across included systematic reviews, which represents a potential limitation of the present umbrella review regarding data duplication and bias. While this is common in areas with well-established bases of evidence [30], the risk of bias due to overlapping data should be carefully considered during the interpretation of the findings.

Substantial redundancy among primary studies was found, especially across reviews incorporating meta-analyses [3,12,13]. This overlap may have led to disproportionate weighting of certain findings, notably those related to root migration, re-intervention, and IAN injury rates, thereby limiting the independence of effect estimates.

#### 4.3.4. Heterogeneity in Inclusion Criteria, Follow-Up, and Outcome Definitions

Marked clinical and methodological heterogeneity was observed across reviews. Inclusion criteria varied with respect to patient populations, surgical techniques, imaging modalities, and study designs. Follow-up durations ranged from 6 to 40 months [8,14], with only a minority of studies extending beyond two years, despite expert recommendations suggesting longer follow-up to capture late complications such as root exposure or infection [29,32,40]. Outcome definitions—particularly those related to re-intervention thresholds, surgical complications, and treatment success—were often inconsistent, limiting the comparability of findings and weakening the foundation for meta-analytical interpretation.

### 4.4. Future Research

This systematic review of systematic reviews provides a comprehensive synthesis of available data, offering a broad perspective on coronectomy outcomes. By consolidating findings from multiple sources, it highlights key trends in patient demographics, complications, re-intervention rates, and long-term success. The inclusion of various studies enhances external validity and allows for the identification of gaps in the literature, such as the lack of standardized imaging protocols, inconsistencies in follow-up durations, and limited documentation on re-intervention outcomes.

However, the study is inherently limited by the quality and heterogeneity of included systematic reviews. Differences in inclusion criteria, surgical techniques, and follow-up protocols across studies introduce variability in reported outcomes, making synthesis across reviews and direct comparisons challenging. Additionally, the lack of detailed patient data, including comorbidities, pharmacological treatments, and long-term post-reintervention outcomes, limits the ability to draw definitive conclusions.

Given the variability in reported outcomes, further research is needed to standardize guidelines and determine the long-term efficacy of coronectomy. While it is an effective option for managing high-risk cases, there is still no universal consensus on its use in clinical practice. Future studies should aim to provide long-term follow-up data and develop protocols to optimize patient outcomes [3,12].

The findings of the present study confirm the need for enhanced documentation of patient characteristics, particularly regarding systemic conditions and pharmacological treatments. As highlighted by Bailey et al. (2020) [38], many studies exclude individuals with systemic conditions, which limits the generalizability of coronectomy findings. This gap in research should be addressed to ensure that coronectomy protocols apply to a broader patient population. Additionally, while the present study recorded a total of 5896 subjects aged 12–95 years, the lack of comprehensive data on gender distribution and medical history remains a concern, as also emphasized by Bernabeu-Mira et al. (2024) [40].

Another area requiring further investigation is preoperative imaging selection. The present study confirmed that panoramic radiography remains the most frequently used imaging tool, despite its limitations in accurately assessing IAN proximity. Bernabeu-Mira et al. (2024) [40] and Simons et al. (2024) [31] emphasized the need for CBCT in high-risk cases, supporting the argument that standardized imaging protocols should be integrated into coronectomy decision-making.

In terms of anatomical assessment, root morphology, distal space, and impaction depth remain underreported in the literature, even though they are crucial for surgical planning [49]. Simons et al. (2024) [31] and Yan et al. (2020) [16] underscored the relevance of these parameters, yet studies, including the present one, have failed to systematically incorporate them into risk stratification models. Future research should prioritize detailed anatomical classification to improve surgical predictability.

Regarding long-term follow-up, the present study’s 6- to 40-month follow-up period aligns with existing research, but studies like Bernabeu-Mira et al. (2024) [40] suggest that longer observational timeframes (beyond five years) are necessary to evaluate late-stage complications, such as root exposure, infection, and periodontal changes. Standardized follow-up intervals should be established to enhance comparability across studies.

The re-intervention rate of 4.29% in the present study highlights the need for a clearer understanding of secondary intervention predictors. While root exposure was the primary cause for re-intervention, Póvoa et al. (2021) [37] reported a lower re-intervention rate (1.13%), suggesting that differences in surgical techniques, patient selection, and follow-up duration significantly impact outcomes. Bailey et al. (2020) [38] emphasized that future research should refine risk factors associated with secondary procedures to reduce unnecessary re-interventions.

Additionally, failure rates and complications after coronectomy require comparisons with those of traditional third molar extractions to better define coronectomy’s role in clinical practice. The present study’s failure rate of 3.48% aligns with the range reported by Simons et al. (2024) [31] and Pang et al. (2024) [33], reinforcing that coronectomy is a relatively safe alternative with a moderate risk of failure. However, more research is needed to establish whether staged extractions (a two-step surgical approach) could further reduce failure and re-intervention rates.

Lastly, data on complications following re-intervention remain scarce. The present study found that no studies documented post-re-intervention pharmacological treatments, mirroring findings from Williams and Tollervey (2016) [45], who stressed the need for comprehensive reporting on secondary procedures. Standardizing data collection on postoperative care, pain management, and long-term healing would improve clinical decision-making and patient outcomes.

## 5. Conclusions

The present umbrella review suggests that coronectomy is a viable alternative in high-risk cases, with a re-intervention rate of 4.45%. Most re-interventions occurred around 10.4 months postoperatively, primarily due to root exposure (16.76%). Root migration was reported in 12.20% of cases, and inferior alveolar nerve injury was rare (0.76%), suggesting a favorable risk profile.

Despite the effectiveness of coronectomy in managing high-risk cases, the heterogeneity in imaging protocols, follow-up durations, and case documentation may have limited the comparability of studies. This underscores the need for standardized patient selection, consistent radiographic assessment, long-term follow-ups, and precise criteria for re-intervention to achieve more predictable outcomes.

While this umbrella review offers valuable insights and highlights consistent trends in coronectomy research, its findings should be interpreted with caution. The heterogeneity, methodological inconsistencies, study overlap, and underreporting of critical variables reduce the strength of its conclusions. Future research should focus on improving patient selection criteria, refining imaging protocols, implementing standardized anatomical and outcome reporting, extending follow-up durations, and including formal assessments of bias. Comparative studies with total extractions will help define the most effective surgical strategies, while clearer guidelines on re-intervention and long-term monitoring will ensure predictable outcomes in third molar management.

## Data Availability

Data are available in the MEDLINE/PubMed, Scopus, Cochrane Library, Web of Science, BioMed Central databases, and PROSPERO register.

## Supplementary Materials

The following supporting information can be downloaded at: https://www.mdpi.com/article/10.3390/jcm14113877/s1, Supplementary File S1: Full Search Strategies; Supplementary File S2: Data extraction.
