# Supplementary material for: Re-Intervention Rate, Timing, and Indications Following Coronectomy of the Mandibular Third Molar: A Systematic Review of Systematic Reviews
Source: J Clin Med. 2025 May 30;14(11):3877. doi: 10.3390/jcm14113877 (PMC12155643; doi:10.3390/jcm14113877)
Supplement: Supplementary file 1 [file jcm-14-03877-s001.zip › jcm-3620930-supplementary.pdf]

Re-Intervention Rate, Timing, and Indications Following Coronectomy of the Mandibular Third Molar: A Systematic Review of Systematic Reviews

Federica Di Spirito *, Mario Caggiano, Alfonso Acerra, Iman Rizki, Grazia Leonetti, Gianluca Allegretti and Massimo Amato

Department of Medicine, Surgery and Dentistry, University of Salerno, Via S. Allende, 84081 Baronissi, Italy; macaggiano@unisa.it (M.C.); alfonso.acerra10@gmail.com (A.A.); i.rizki@studenti.unisa.it (I.R.); dottg.leonetti@gmail.com (G.L.); dott.allegrettigianluca@gmail.com (G.A.); mamato@unisa.it (M.A.)

* Correspondence: fdispirito@unisa.it

Supplementary File S1 – Full Search Strategies

Database	Date of Search	Search String	Filters
**MEDLINE/PubMed**	30 Dec 2024	("TITLE-ABS-KEY"[All Fields] AND ("coronectomy"[All Fields] OR "partial odontectomy"[All Fields] OR "intentional root retention"[All Fields]) AND "TITLE-ABS-KEY"[All Fields]) AND ("third molar"[All Fields] OR "inferior molar"[All Fields] OR "wisdom tooth"[All Fields] OR "wisdom teeth"[All Fields])	Article Type: Systematic Review; Language: English
**Scopus**	30 Dec 2024	(TITLE-ABS-KEY ((coronectomy OR "partial odontectomy" OR "intentional root retention" OR "root re-tention technique")) AND TITLE-ABS-KEY (("third molar" OR "inferior molar" OR "wisdom tooth" OR "wisdom teeth")))	Document Type: Review; Language: English
**Web of Science**	30 Dec 2024	ALL=((coronectomy OR "partial odontectomy" OR "intentional root retention" OR "root retention technique") AND ("third molar" OR "wisdom tooth" OR "wisdom teeth" OR "inferior molar"))	Document Type: Review; Language: English
**BioMed Central**	30 Dec 2024	(coronectomy OR "partial odontectomy" OR "intentional root retention" OR "root retention technique") AND ("third molar" OR "wisdom tooth" OR "wisdom teeth" OR "inferior molar")	No filters applied
**Cochrane Library**	30 Dec 2024	((coronectomy OR "partial odontectomy" OR "intentional root retention" OR "root retention technique") AND ("third molar" OR "wisdom tooth" OR "wisdom teeth" OR "inferior molar")):ti,ab,kw	No filters applied
**PROSPERO**	30 Dec 2024	coronectomy OR "partial odontectomy" OR "intentional root retention" OR "root retention technique" AND "third molar" OR "wisdom tooth" OR "wisdom teeth" OR "inferior molar"	Status: Published

Supplementary File S2 – Data Extraction

The following data were extracted from each systematic review, regardless of whether it included a meta-analysis:

- Studies characteristics:

- First Author, year of publication, reference, journal, number and design of included studies, quality assessment, meta-analysis (yes/no), funding (if any);

- Population characteristics:
  - Sample size, mean age (range), gender ratio (M/F), comorbidities, and ongoing pharmacological treatments of the study population of each systematic review;
- Inferior third molar characteristics:
  - Third molar treated with coronectomy: total number; side (right n…/…%, left n…/…%), root morphology; angulation (mesioangular/vertical/horizontal/distoangular); distal space: (es.: Pell & Gregory I/II/III) [28]; depth: (es.: mm / Pell & Gregory A/B/C) [28]; proximity to anatomical structures; degree of impaction: erupted/semierupted/partial bony impaction/intraosseous; presence/absence of the second molar (n…/...%);
- Coronectomy:
  - coronectomy: pre-operative radiography (type and number), indications (type and number); post-operative pharmacological treatment(s): (type and duration); complications (IANI, t-IANI, p-IANI, LNI, Pain, Infections, Alveolar osteitis, Root migration, root exposure), follow-up (months), failure (tooth extracted), patient-related outcomes;
- Re-intervention rate, timing and collection:
  - re-intervention: rate: (n…./...%); timing (months); indications - root migration/exposure (n…/…%), infection (n…/…%), pain (n…/…%), residual enamel (n…/…%), palpable root (n…/…%), incomplete healing (n…/…%), periodontal disease (n…/…%), orthodontic procedure (n…/…%), hyperplasia distally to second molar (n…/…%), root moved during procedure (n…/…%), pharmacological treatments after re-intervention, patient-related outcomes.
